# Mucosal Invasion, but Not Incomplete Excision, Has Negative Impact on Long-Term Survival in Patients With Extramammary Paget’s Disease

**DOI:** 10.3389/fonc.2021.642919

**Published:** 2021-04-15

**Authors:** Hiroki Hashimoto, Yumiko Kaku-Ito, Masutaka Furue, Takamichi Ito

**Affiliations:** Department of Dermatology, Graduate School of Medical Sciences, Kyushu University, Fukuoka, Japan

**Keywords:** extramammary Paget’s disease, mucosal invasion, surgery, prognostic factor, invasive surgery, radical surgery

## Abstract

**Background:**

Extramammary Paget’s disease (EMPD) sometimes spreads from the skin to mucosal areas, and curative surgical excision of these areas is challenging. The aim of this study is to analyze the impact of mucosal involvement and surgical treatment on the survival of patients with EMPD.

**Methods:**

We conducted a retrospective review of 217 patients with EMPD. We also assessed the associations between tumor involvement in boundary areas (anal canal, external urethral meatus, vaginal introitus), prognostic factors, and survival in 198 patients treated with curative surgery.

**Results:**

Of 217 patients, 75 (34.6%) had mucosal boundary area involvement. Lesions in these areas were associated with frequent lymphovascular invasion (*p* = 0.042), lymph node metastasis (*p* = 0.0002), incomplete excision (*p* < 0.0001), and locoregional recurrence (*p* < 0.0001). Boundary area involvement was an independent prognostic factor associated with disease-specific survival, per multivariate analysis (HR: 11.87, *p* = 0.027). Incomplete excision was not significantly correlated with disease-specific survival (HR: 1.05, *p* = 0.96).

**Conclusion:**

Boundary area tumor involvement was a major risk factor for incomplete excision, local recurrence, and poor survival outcomes. However, incomplete removal of primary tumors was not significantly associated with poor prognosis. A less invasive surgical approach for preserving anogenital and urinary functions may be acceptable as the first-line treatment for resectable EMPD.

## Introduction

Extramammary Paget’s disease (EMPD) is a rare neoplastic condition (1). It commonly affects areas rich in apocrine sweat glands, including the vulva, perineal area, perianal area, scrotal area, and penile skin (1, 2). EMPD typically affects Caucasian females and Asian males older than 60 years (3–7). Most EMPD tumors are restricted to the epidermis as *in situ* lesions, and they are associated with good prognosis because of their slow-growing nature (1, 8). However, approximately 15–40% of EMPD lesions display dermal invasion, which is known as invasive EMPD, and this increases the risk of lymph node and distant metastasis (2, 4). Management is notoriously complicated, and the recurrence rate is high (15–61%) despite aggressive surgeries (9–12).

Several prognostic factors regarding primary tumors have been reported, including tumor thickness (13, 14), level of tumor invasion (15–18), lymphovascular invasion (8, 17, 19), and perianal location (13, 20–22). Ohara et al. (8) recently conducted a multicenter analysis of 301 invasive EMPD cases, and they proposed a new tumor, node, and metastasis (TNM) classification and staging system in which the T category was determined based on tumor thickness and lymphovascular invasion. The Japanese Skin Cancer Society is currently proposing the use of this EMPD-specific TNM classification and staging system. However, the classification is still tentative.

EMPD lesions sometimes spread from the skin to mucosal areas *via* boundary areas (anal canal, external urethral meatus, vaginal introitus) and deep toward internal organs (rectum, uterus, urinary bladder). Curative surgical excision of lesions in boundary areas is challenging since radical excision impairs organ functions and requires additional functional reconstruction (colostomy, etc.). To preserve organ function, surgical margins are determined at specific sites (e.g., dentate line) regardless of tumor spread, but it can be difficult to maintain sufficient surgical margins at these sites. Perianal lesions indicate poor prognosis partly due to difficult total excision (20). A recent report suggested frequent incomplete excision in cases of EMPD with mucosal involvement (23). However, the prognostic impact of mucosal involvement has not been elucidated.

In this study, we reviewed the data of 217 EMPD patients in our institution over a 23-year period. We showed that lesions involving boundary areas were associated with high risk for poor survival outcomes, regardless of whether complete surgical removal was achieved, and that incomplete excision of EMPD did not affect patient outcomes. We also aimed to verify the newly proposed EMPD-specific TNM staging system (8).

## Materials and Methods

### Patients

This retrospective review was conducted according to the guidelines of the Declaration of Helsinki. This study was approved by the Ethics Committee of Kyushu University Hospital (30–363; November 27, 2018). We retrieved the data of 217 patients with primary EMPD lesions. These patients were treated at the Department of Dermatology of Kyushu University in Fukuoka, Japan, between January 1997 and October 2020. At least three experienced dermatopathologists confirmed the diagnosis. Patients with secondary EMPD, which involved direct invasion from visceral organs, were carefully excluded.

The following data on all patients were retrieved from our prospectively maintained databank and then analyzed: demographic data (sex, age at initial presentation), clinical data (tumor site, primary lesion size), and histopathological data obtained *via* hematoxylin and eosin staining (tumor thickness [measured to the second decimal place, as per the latest melanoma classification guidelines of the American Joint Committee on Cancer] (24), lymphovascular invasion). For patients with two or more primary lesions, we recorded the greatest tumor thickness and the total tumor size. Tumor thickness was measured from the total excised specimen. For cases without total excision, tumor thickness was calculated from biopsy specimens. In situ lesions on biopsy were further confirmed by clinical findings (lack of erosions, ulcerations, formation of nodules). Involvement of mucosal boundary areas (anal canal, external urethral meatus, vaginal introitus) was recorded from clinicopathological data. Lymph node metastasis was primarily determined by histopathology. Patients who had lymphadenopathy detected by physical examination or imaging studies (ultrasonography, computed tomography [CT], and/or positron emission tomography with computed tomography [PET/CT]) were also considered to have metastasis. The N category was defined according to the classification system proposed by Ohara et al. (8): N0, no lymph node metastasis; N1, metastasis involving one lymph node; and N2, metastasis involving two or more lymph nodes. Distant metastasis was determined by using imaging studies (ultrasonography, chest X-ray, CT, and/or PET/CT). Lymph node metastasis beyond the regional lymphatic basin was also classified as distant metastasis. For the M category, M0 indicated no distant metastasis, and M1 indicated distant metastasis (8).

### Mucosal Boundary Area Involvement and Surgical Outcomes

Next, the data of patients treated with curative surgery were collected. Patients were divided into two groups, that is, with or without involvement of mucosal boundary areas, as involvement of these areas influences surgical strategies. In addition to the data mentioned above, we compared data pertaining to surgical treatments and outcomes, including surgical margin, margin status after surgery (complete or incomplete excision), local recurrence, and new regional lymph node metastasis after initial treatment, between these two groups. Complete excision was defined as complete removal of the primary tumor with histopathologically negative margins and complete dissection of regional lymph nodes (if lymph node metastases were present). Patients with distant metastases at surgery were excluded when comparing surgical outcomes. Reconstruction of skin/mucosal defects was performed by using simple sutures, skin grafting, or musculocutaneous flaps, as appropriate.

### Follow-Up

The patients were monitored by physical examination every 3–6 months and imaging studies (ultrasonography, chest X-ray, and/or CT). Survival data, including time of locoregional and distant recurrence, survival length, and cause of death, were recorded. The median follow-up period was 61.4 months (range: 2.0–264.7 months). By the last follow-up, 164 patients were alive, 20 died of EMPD, and 33 died of other causes.

### Statistical Analysis

All statistical analyses were performed by using JMP version 14.2 (SAS Institute, Cary, NC, USA). The χ^2^ test or Fisher’s exact test and Mann-Whitney U test were used for analysis of categorical variables and continuous variables, respectively. We used the Kaplan-Meier method to evaluate disease-specific survival (DSS), and we compared survival curves by using the log-rank test. DSS was calculated from the date of the first histological examination to the date of death due to EMPD or the last follow-up prior to October 31, 2020. Data on patients who did not die were censored on October 31, 2020. Data on patients who died of other causes were censored at the time of death. The associations between clinical and histopathological factors and DSS were determined by using a multivariate Cox proportional hazards regression model. Probability values less than 0.05 were regarded as statistically significant.

## Results

### Clinicopathological Data of the Study Cohort

The demographic and clinical data of the 217 patients with primary EMPD are shown in **Table 1**. All patients were Japanese, with a mean age of 72.9 years (range: 34–95 years). There were 130 male patients (59.9%) and 87 female patients (40.1%). Tumors were predominantly localized in the genital area (83.9%), followed by the perianal area (4.1%), then the axillary area (2.3%). Multiple lesions or tumors spreading over two areas were seen in 21 patients (9.7%). There were 95 patients (44.4%) with small primary lesions (< 25 cm^2^) and 119 (55.6%) with large lesions (≥ 25 cm^2^). A total of 109 patients (50.2%) had tumors in situ. Tumor thickness was stratified as ≤ 1 mm, 1–4 mm, or > 4 mm for invasive tumors. There were 38 patients (17.5%) with tumors ≤ 1 mm, 45 (20.7%) with tumors 1–4 mm, and 19 (8.8%) with tumors > 4 mm. Lymphovascular invasion was observed in 14 patients (6.5%); lymphovascular invasion was not evident in 203 patients (93.5%). A total of 75 patients (34.6%) exhibited boundary area involvement. Regional lymph node metastasis was found in 27 patients (12.4%). Seven patients (3.2%) had one metastatic lymph node, and 20 (9.2%) had two or more. Distant metastasis was observed in six patients (2.8%). Data on primary lesion size and tumor thickness were unavailable for three and six patients, respectively.

**Table 1:** Demographics and clinical data of all 217 patients.

Parameter	n (%)
Sex	
Male Female	130 (59.9) 87 (40.1)
Age (years)	
Mean ± SD Median (range)	72.9 ± 10.0 73 (34-95)
Tumor site	
Genital area only Perianal area only Axillary area only Genital + perianal areas Genital + axillary areas Other areas	182 (83.9) 9 (4.1) 5 (2.3) 13 (6.0) 5 (2.3) 3 (1.4)
Primary lesion size (cm^2^)	
<25 ≥25 Unknown	95 (44.4) 119 (55.6) 3 (0.4)
Tumor thickness (mm)	
In situ ≤1 1-4 >4 Unknown	109 (50.2) 38 (17.5) 45 (20.7) 19 (8.8) 6 (2.8)
Lymphovascular invasion	
Present Absent	14 (6.5) 203 (93.5)
Boundary area involvement	
Present Absent	75 (34.6) 142 (65.4)
Metastasis	
Regional lymph node metastasis	
N0 N1 N2	190 (87.6) 7 (3.2) 20 (9.2)
Distant metastasis	
M0 M1	211 (97.2) 6 (2.8)

SD, standard deviation.

### Treatment, Locoregional Recurrence, and Distant Metastasis

A total of 204 patients (94.0%) underwent surgical excision for primary lesions. Of these patients, 200 underwent curative excision with wide margins (0.5–5.0 cm), typically after mapping biopsy, and four underwent palliative surgery. Surgical margins were positive in 46 of these 204 patients (22.5%). Additional excision was performed in seven of these 46 patients. A total of 13 patients (6.0%) with disseminated metastasis or complications or who were unable to give consent for surgical excision received the following alternative treatments, alone or in combination: topical imiquimod cream (n = 3), topical 5-fluorouracil ointment (n = 3), cryotherapy (n = 2), photodynamic therapy (n = 1), radiation therapy (n = 5), or systemic chemotherapy (n = 4). Only two patients received palliative care as the primary treatment. There were 33 patients without lymphadenopathy who underwent sentinel lymph node biopsy (SLNB); eight of them (24.2%) were positive. There were 19 patients with lymphadenopathy who underwent swollen lymph node biopsy; nine of them (47.4%) had confirmed metastasis. Completion lymph node dissection (CLND) was performed in 18 patients (8.3%). Systemic chemotherapy/targeted therapy was performed in six patients (2.8%). Radiation therapy was performed in seven patients (3.2%). A summary of the initial treatments is available in **Supplementary Table 1**.

Of 200 patients who underwent curative excision with wide margins, 13 patients had local recurrence during the follow-up period. They underwent wide surgical excision (n = 9), radiation therapy (n = 2), or treatment with topical imiquimod cream (n = 2). The details of the 13 patients with local recurrence are shown in **Supplementary Table 2**. Regional lymph node metastasis or distant metastasis (distant lymph node, lung, liver, brain, or bone metastasis) occurred for the first time in 18 patients during the follow-up period, and 13 of these patients underwent CLND, systemic chemotherapy/targeted therapy, or radiation therapy (alone or in combination).

### Stage Classification and Disease-Specific Survival: Corroboration of the Newly Proposed TNM Staging System

**Figure 1:**
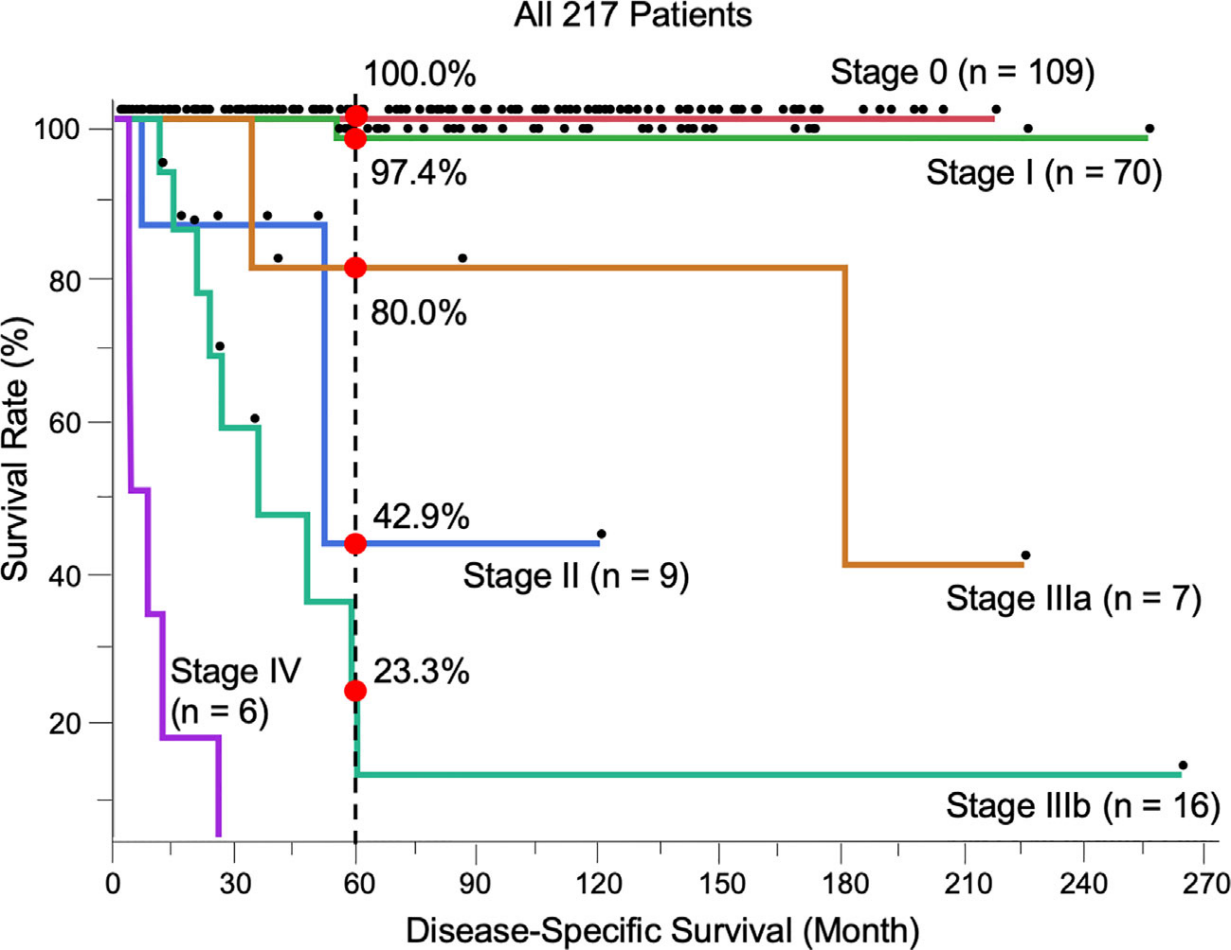
Kaplan-Meier disease-specific survival curves of all 217 patients stratified by TNM stage. The 5-year survival was 100.0% (Stage 0, n = 109), 97.4% (I, n = 70), 42.9% (II, n = 9), 80.0% (IIIa, n = 7), 23.3% (IIIb, n = 16), and 0.0% (IV, n = 6). The log-rank test showed the results of survival as follows; 0 vs I, *p* = 0.17; I vs II, *p* < 0.0001; I vs IIIa, *p* = 0.034; I vs IIIb, *p* < 0.0001; II vs IIIa, *p* = 0.47; II vs IIIb, *p* = 0.24; IIIa vs IIIb, *p* = 0.066; 0 vs. IV, *p* < 0.0001; I vs. IV, *p* < 0.0001; II vs. IV, *p* = 0.0027; IIIa vs. IV, *p* = 0.0003; IIIb vs. IV, *p* < 0.0001.

Most patients were stage 0 (T0N0M0) (n = 109, 50.2%), followed by stage I (T1N0M0) (n = 70, 32.3%), stage II (T2N0M0) (n = 9, 4.1%), stage IIIa (TanyN1M0) (n = 7, 3.2%), stage IIIb (TanyN2M0) (n = 16, 7.4%), and stage IV (TanyNanyM1) (n = 6, 2.8%). The 5-year DSS of each stage was 100.0%, 97.4%, 42.9%, 80.0%, 23.3%, and 0.0%, respectively. The prognosis between stages I and II, classified by tumor thickness of invasive EMPD without remote regional lymph node or distant metastasis, showed a significant difference (*p* < 0.0001). All patients with distant metastasis (stage IV) died within 5 years, and the survival rate was significantly different from that of all other stages (0 vs. IV, *p* < 0.0001; I vs. IV, *p* < 0.0001; II vs. IV, *p* = 0.0027; IIIa vs. IV, *p* = 0.0003; IIIb vs. IV, *p* < 0.0001). No significant difference was found between stages IIIa and IIIb, classified by the number of lymph node metastases (*p* = 0.066). There were significant differences in survival between stages I and IIIa (*p* = 0.034) and stages I and IIIb (*p* < 0.0001). The survival rate of stages II was opposite that of patients in stage IIIa, although there was no significant difference (*p* = 0.47). The Kaplan-Meier DSS curves of patients stratified by TNM stage are shown in **Figure 1**.

### Characteristics of Patients Treated With Curative Surgery

Next, the data of 198 patients treated with curative surgery were analyzed to assess the associations between mucosal boundary area involvement and prognostic factors. Patients with distant metastasis (stage IV) were excluded from this analysis. There were 65 patients (32.8%) with boundary area involvement and 133 (67.2%) without.

The demographic and clinicopathological data of each group are listed in **Table 2**. Patients with involvement of boundary areas were mostly female (*p* < 0.0001), and the location was most frequently the perianal area (*p* = 0.0018). Tumor size showed no significant difference between the two groups (*p* = 0.29). Histopathologically, patients with boundary area involvement tended to have thicker tumors in invasive EMPD (*in situ* vs. ≤ 4 mm, *p* = 0.65; *in situ* vs. > 4 mm, *p* = 0.040; ≤ 4 mm vs. > 4 mm, *p* = 0.077). Lymphovascular invasion was more frequently observed in patients with involvement of boundary areas (*p* = 0.042). Patients with boundary area involvement had more advanced primary tumors. The rate of regional lymph node metastasis in patients with boundary area involvement was statistically higher than in patients without boundary area involvement (*p* = 0.0002). In each group, patients were classified in accordance with the TNM staging system. Patients with involvement of boundary areas tended to be classified with advanced TNM stages.

**Table 2:** Demographics and clinical data of the 198 patients treated with curative surgery.

Parameter	Involvement of mucosal boundary areas		*P*-value*
	Present (n = 65)	Absent (n = 133)	
Sex			
Male Female	16 (24.6%) 49 (75.4%)	105 (78.9%) 28 (21.1%)	**<0.0001**
Age (year)			
Mean ± SD	69.7 ± 10.3	73.5 ± 9.12	**0.0091**
Tumor site			
Perianal area Other areas	12 (18.5%) 53 (81.5%)	5 (3.8%) 128 (96.2%)	**0.0018**
Primary lesion size (cm^2^)			
<25 ≥25	26 (40.0%) 39 (60.0%)	64 (48.1%) 69 (51.9%)	0.29
Tumor thickness (mm)			
In situ ≤4 >4	30 (46.2%) 26 (40.0%) 9 (13.8%)	72 (54.1%) 54 (40.6%) 7 (5.3%)	0.12^†^
Lymphovascular invasion			
Present Absent	7 (10.8%) 58 (89.2%)	4 (3.0%) 129 (97.0%)	**0.042**
Regional LN metastasis			
Present Absent	13 (20.0%) 52 (80.0%)	4 (3.0%) 129 (97.0%)	**0.0002**
Number of regional LN metastases			
1 2 or more	4 (30.8%) 9 (69.2%)	3 (75.0%) 1 (25.0%)	0.25
TNM stage			
0 I II IIIa IIIb	30 (46.2%) 20 (30.8%) 2 (3.1%) 4 (6.2%) 9 (13.9%)	72 (54.1%) 50 (37.6%) 7 (5.3%) 3 (2.3%) 1 (0.8%)	**0.0014**
Local recurrence			
Present Absent	12 (18.5%) 53 (71.5%)	0 (0.0%) 133 (100.0%)	**<0.0001**
Follow-up period (month)			
Mean ± SD Median (range)	82.8 ± 64.0 58.2 (7.2–256.5)	83.7 ± 57.4 78.9 (2.0–264.7)	0.73

Significant values are shown in boldface.

*Mann-Whitney U tests were used for continuous variables, and χ^2^ or Fisher’s exact tests were used for categorical variables.

^†^In situ vs. ≤ 4 mm, p = 0.65; in situ vs. > 4 mm, p = 0.040; ≤ 4 mm vs. > 4 mm, p = 0.077.

SD, standard deviation; LN, lymph node; TNM, tumor, node, metastasis.

Twelve patients had local recurrence during the follow-up period, and all of them had involvement of boundary areas. They underwent wide surgical excision (n = 9), radiation therapy (n = 1), or treatment with topical imiquimod cream (n = 2). The details of the patients with local recurrence are shown in **Supplementary Table 2**.

### Initial Treatment of Patients Treated With Curative Surgery: Boundary Area Involvement as a Risk Factor for Incomplete Excision

The initial treatment patterns of these 198 patients, who were divided into two groups based on boundary area involvement, are summarized in **Table 3**.

**Table 3:** Initial treatment of the 198 patients treated with curative surgery.

Treatment		Involvement of boundary areas		P-value*
		Present (n = 65)	Absent (n = 133)	
For primary lesions	Surgical margin (cm)			
	Mean ± SD	1.56 ± 0.84	1.72 ± 0.84	0.18
	Surgical margin status			
	Positive Negative	34 (52.3%) 31 (47.7%)	8 (6.0%) 125 (94.0%)	**<0.0001**
	Additional excision			
	Done Not done	6 (17.7%) 28 (82.3%)	1 (12.5%) 7 (87.5%)	1.00
For regional LNs	SLNB			
	Done Not done SLNB LN metastasis present No LN metastasis Biopsy of lymphadenopathy Done Not done Biopsy of lymphadenopathy LN metastasis present No LN metastasis CLND Done Not done	8 (12.3%) 57 (87.7%) 5 (62.5%) 3 (37.5%) 8 (12.3%) 57 (87.7%) 5 (62.5%) 3 (37.5%) 13 (20.0%) 52 (80.0%)	24 (18.1%) 109 (81.9%) 2 (8.3%) 22 (91.7%) 8 (6.0%) 125 (94.0%) 1 (12.5%) 7 (87.5%) 4 (3.0%) 129 (97.0%)	0.41 **0.0048** 0.16 0.12 **0.0002**
Overall	Complete excision^†^			
	Complete Incomplete	37 (56.9%) 28 (43.1%)	126 (94.7%) 7 (5.3%)	**<0.0001**
Adjuvant therapy	Chemotherapy	0 (0.0%)	1 (0.75%)	1.00
	Radiation therapy	1 (0.75%)	0 (0.0%)	1.00

Significant values are shown in boldface.

*Mann-Whitney U tests were used for continuous variables, and Fisher’s exact tests were used for categorical variables.

^†^Complete excision was defined as complete removal of the primary tumor with histopathologically negative margins and complete dissection of regional lymph nodes (if lymph node metastases were present).

SD, standard deviation; LN, lymph node; SLNB, sentinel lymph node biopsy; CLND, completion lymph node dissection.

For primary tumor excision, the distance of the surgical margin showed no significant difference in the two groups (mean: 1.56 cm vs. 1.72 cm, *p* = 0.18). Surgical margins were positive in 42 of the 198 patients (21.2%). The positive site was predominantly at the mucosal side (n = 30), followed by the skin side (n = 8), and then both the mucosal and skin sides (n = 4). The positive surgical margin rate was significantly higher in patients with boundary area involvement than in patients without boundary area involvement (*p* < 0.0001). Additional excision was performed in seven of the 42 patients with positive surgical margins (six patients with additional mucosal excision and one with additional skin excision), and all seven of these patients were confirmed to have negative surgical margins. Only three patients underwent colostomy or urinary diversion. There was no significant difference in the rate of SLNB performed (*p* = 0.41). However, the rate of metastasis in SLNB cases was significantly different between the two groups (*p* = 0.0048). The rate of metastasis in lymphadenopathy cases was not significantly different between the two groups (*p* = 0.12). CLND was performed in 13 patients with boundary area involvement and four patients without boundary area involvement (*p* = 0.0002). Curative excision was completed in 37 patients with boundary area involvement (56.9%) and 126 patients without boundary area involvement (94.7%) (*p* < 0.0001). All incomplete excisions were for primary tumors. There were no patients with incomplete removal of regional lymph nodes. Five patients among 35 patients with incomplete excision (14.3%) experienced local recurrence (**Supplementary Table 2**).

### Factors Associated With Disease-Specific Survival of Patients Treated With Curative Surgery: Negative Impact of Boundary Area Involvement on Long-Term Survival

**Figure 2:**
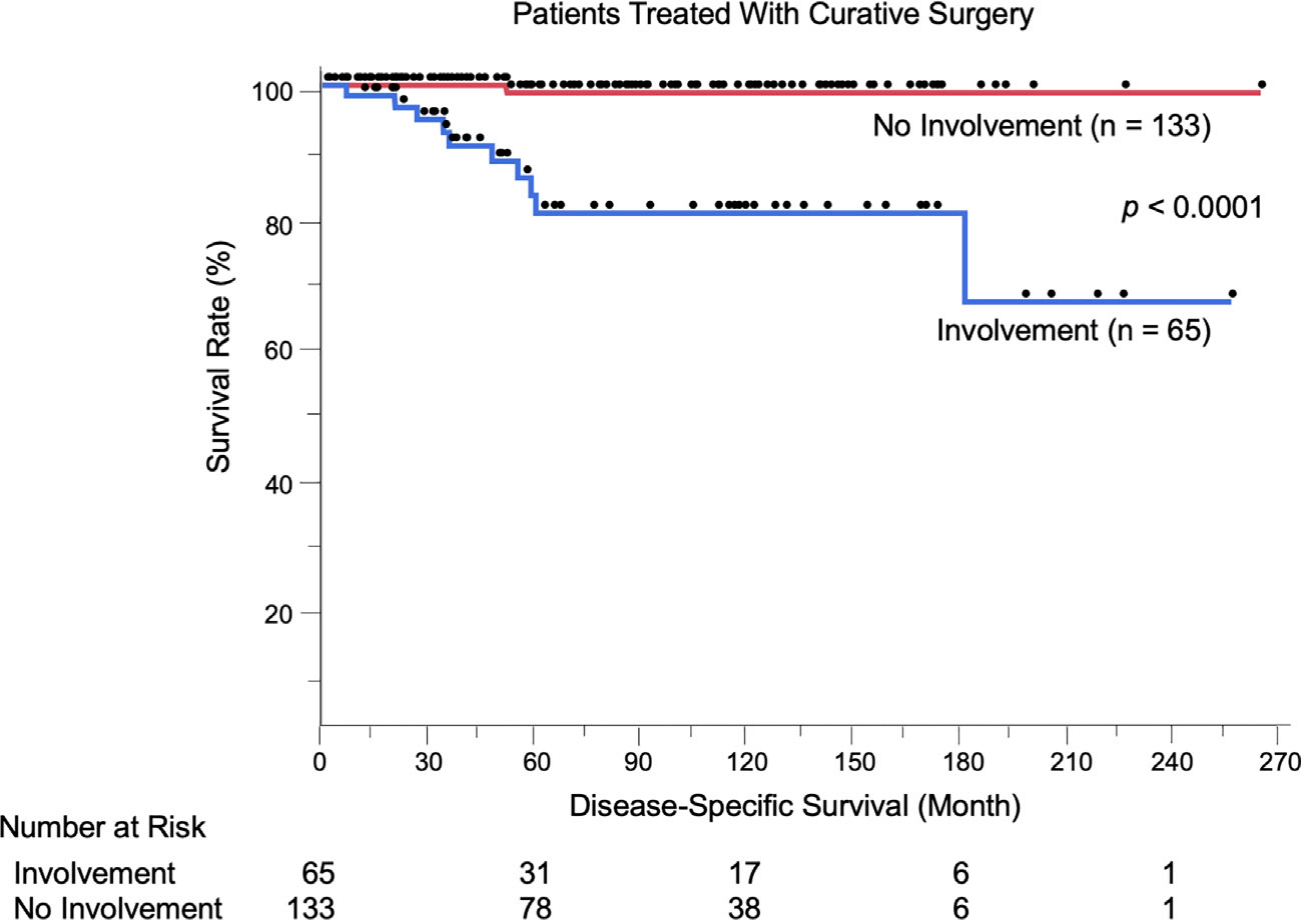
Kaplan-Meier disease-specific survival curves of the 198 patients treated with curative surgery stratified by boundary area involvement. Patients with EMPD lesions in boundary areas had significantly shortened their survival (*p* < 0.0001). The number at risk is also shown.

**Figure 3:**
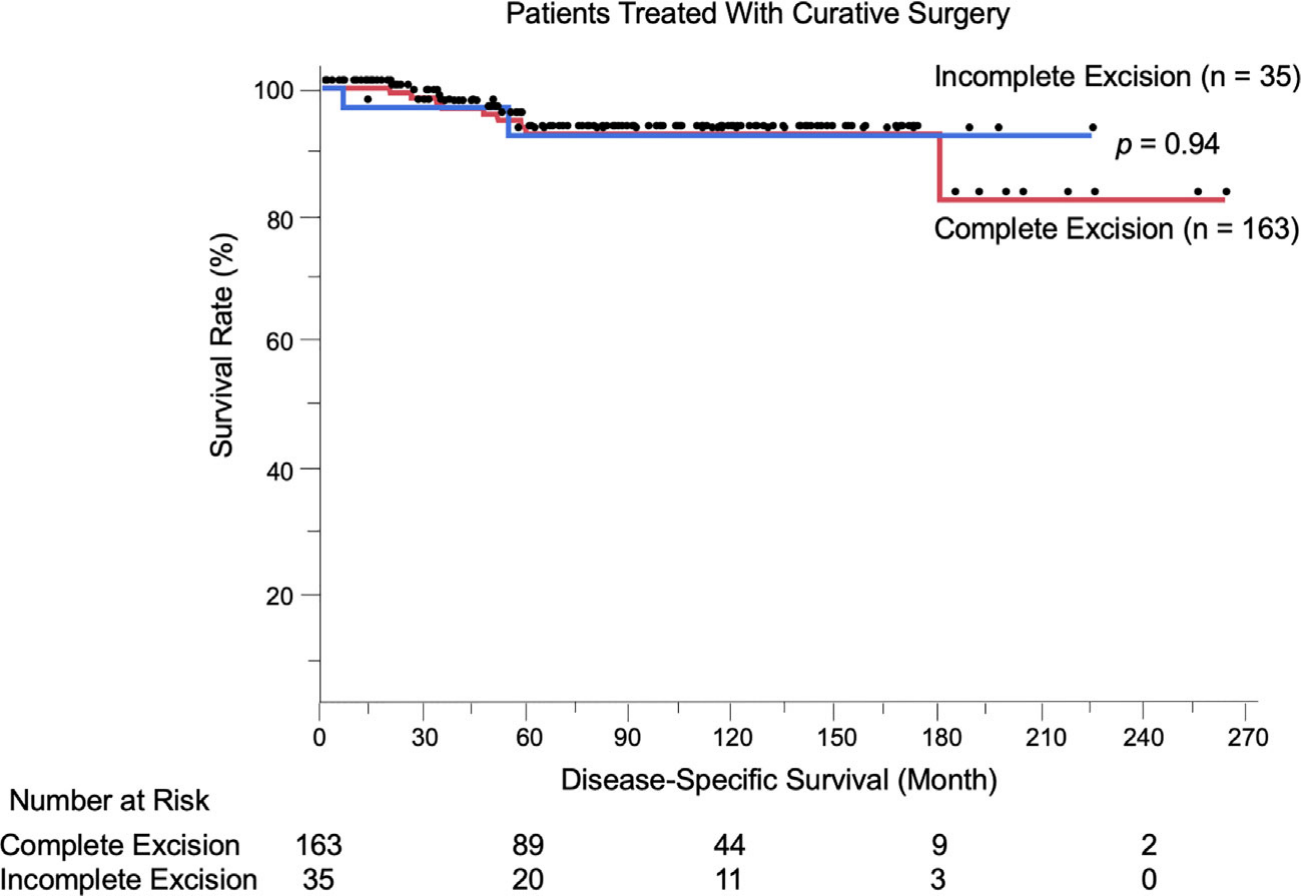
Kaplan-Meier disease-specific survival curves of the 198 patients treated with curative surgery stratified by achievement of complete excision. Incomplete excision was not correlated with worse survival compared to complete excision (*p* = 0.94).The number at risk is also shown.

We evaluated the possible clinical and histopathological factors associated with DSS in the 198 patients treated with curative surgery by using a multivariate Cox proportional hazards regression model. The following factors were included as explanatory variables: sex, age, tumor site, tumor thickness, boundary area involvement, complete excision, and regional lymph node metastasis. The results are listed in **Table 4**. Univariate analysis results revealed that tumor thickness > 4 mm, boundary area involvement, and regional lymph node metastasis were statistically significant factors for poor survival. Multivariate analysis results showed that tumor thickness > 4 mm (HR: 7.23, *p* = 0.0037), boundary area involvement (HR: 11.87, *p* = 0.027), and regional lymph node metastasis (HR: 27.91, *p* = 0.031) were also statistically independent factors associated with DSS. Incomplete excision was not significantly correlated with survival (HR: 1.05, *p* = 0.96). The Kaplan-Meier curves of patients stratified by boundary area involvement and achievement of complete excision are shown in **Figures 2**, **3**.

**Table 4:** Multivariate Cox proportional hazard analyses for disease-specific survival.

Variable	Univariate analysis			Multivariate analysis		
	HR	95% CI	*P*-value	HR	95% CI	*P*-value
Sex, male	1.78	0.47-6.72	0.39	0.26	0.012-5.42	0.38
Age (year)^†^	1.01	0.92-1.05	0.49	1.05	0.97-1.14	0.24
Perianal lesion	1.11	0.14-8.72	0.92	1.53	0.13-16.90	0.73
Tumor thickness > 4 mm	30.56	8.73-109.94	**<0.0001**	7.23	1.13-46.19	**0.037**
Boundary area involvement	21.13	2.70-165.60	**0.0037**	11.87	1.32-106.73	**0.027**
Incomplete excision	0.94	0.20-4.38	0.94	1.05	0.16-6.74	0.96
Regional LN metastasis	36.60	9.51-140.92	**<0.0001**	27.91	1.35-576.63	**0.031**

Significant values are shown in boldface.

^†^Continuous variable.

HR, hazard ratio; CI, confidence interval; LN, lymph node.

As an additional analysis, these possible prognostic factors were evaluated in the 65 patients with boundary area involvement by using a multivariate analysis for DSS. The results revealed that incomplete excision was not significantly correlated with survival (HR: 3.11, *p* = 0.34). The detailed data are available in **Supplementary Table 3**.

## Discussion

Complete surgical tumor removal is the treatment of choice for resectable EMPD. Due to the slow-growing nature of this kind of tumor, nearly 90% of the patients at our hospital show no lymph node or distant metastasis. Treatment strategies for primary lesions are therefore key for curing this disease in these patients. EMPD lesions are most likely to arise in the anogenital area, sometimes extending toward visceral organs *via* boundary areas (anal canal, external urethral meatus, vaginal introitus). When tumors involve these boundary areas, surgeons are forced to choose whether radical surgical excision with extensive reconstruction should be performed or whether less invasive surgery should be performed to preserve defecation and urination functions. This choice is challenging, as most EMPD patients are elderly, and radical surgery impairs patients’ quality of life. The latter choice is often chosen in our institute after deep discussion with patients and their families, unless the tumors are invasive (with nodule formation, etc.) in boundary areas. Reconstruction of skin/mucosal defects is typically accomplished by using simple sutures or split-skin grafting. One of the aims of this study was to evaluate the reasonability of this kind of surgery. We retrospectively summarized 23 years of experience treating 217 patients with EMPD and assessed their outcomes. This is one of the largest studies conducted at a single institute, and we identified several important findings.

We showed for the first time that patients with EMPD lesions in boundary areas had significantly shortened DSS compared to other patients (*p* < 0.0001, **Figure 2**). This was corroborated by the results of multivariate analyses, which were adjusted by some known prognostic factors (HR: 11.87, 95% CI: 1.32–106.73, *p* = 0.027). Representative prognostic factors of primary tumors include nodule formation (14, 25), tumor thickness (8, 13, 14), level of tumor invasion (15–18), lymphovascular invasion (8, 17, 19), perianal location (13, 20–22), and vaginal location (26). Human epidermal growth factor receptor 2/neu (27–29) and nectin cell adhesion molecule 4 (30) expression are other factors associated with tumor recurrence and DSS, respectively. We previously evaluated the efficacy of mapping biopsy and surgical treatment of EMPD, and we found a high tumor-positive rate of surgical margins in EMPD lesions with mucosal boundary area involvement (19/36, 52.8%) (23). This high positive rate may be due to difficulty both in delineating tumor borders and in setting sufficient surgical margins in these areas. In the current study, the positive rate was similar to our previous one (34/65, 52.3%). Some factors were associated with the presence of boundary area involvement. Female patients more frequently had boundary and perianal lesions compared to male patients (data not shown) since female anogenital areas are close to boundary areas. Other factors included thicker tumors, the presence of lymphovascular invasion, and lymph node metastasis, suggesting that advanced EMPD lesions are likely to extend to boundary areas. In this study, 12 patients experienced local recurrence of primary lesions, and all had boundary lesions.

Of note, among the 198 patients treated with curative surgery, incomplete excision of primary tumors was not correlated with worse DSS compared to complete removal (*p* = 0.94). Similarly, when analyzing the patients with boundary area involvement (n = 65), incomplete excision was not a poor prognostic factor (*p* = 0.34 per Cox multivariate analysis). Furthermore, only five patients among 35 patients with incomplete excision (14.3%) experienced local recurrence. Most of the patients with the disease were elderly (mean age: 72.9 years), and among the 53 patients who died during the follow-up period, EMPD was the direct cause only in 20 patients (37.7%); the other 33 patients (62.3%) died of other causes. These results raise an important question: is it always necessary to pursue negative margins in primary EMPD? Previous studies have reported no correlation between positive surgical margins and local recurrence in vulvar EMPD (9–11, 31, 32). Nasioudis et al. (6) conducted a large database study and reported that the presence of positive surgical margins was not associated with overall survival. Correlations between surgical margins and patient survival have been controversial, and the current study offered new insights into this issue. Furthermore, some radical surgical procedures (proctectomy, urethrectomy, total cystectomy) are accompanied by simultaneous creation of colostomy and urinary diversions, which can lead to troublesome complications (33–36). Formijne Jonkers et al. (37) reported that 82% of patients who underwent creation of an intestinal stoma experienced one or more stoma-related complications within 1 year. Radical surgeries with creation of colostomy or urinary diversions deteriorate patients’ organ functions, as well as patients’ quality of life (33, 38–40). In our cohort, only three of 75 patients (4.0%) with boundary area involvement underwent colostomy or urinary diversion. Whereas lesions in boundary areas had increased risks of incomplete excision and local recurrence, these lesions were also associated with advanced tumor status (thicker tumors, frequent lymphovascular invasion, and lymph node metastasis). Most localized EMPD lesions were unaggressive, with high 5-year survival rates (100% in stage 0 and 97.4% in stage I). Collectively, the less invasive approach we performed (preserving anorectal and urinary functions) may be a reasonable treatment choice for patients with EMPD.

Another interesting finding was that patient survival in this study fit well with the newly proposed TNM staging system (8). Although TNM staging is crucial in cancer treatment, no widely accepted staging system specific for EMPD has been established due to the rarity of the disease. In this study, we classified patients in accordance with the newly proposed, EMPD-specific TNM staging system (8) and assessed its validity. The T category (classified by tumor thickness and lymphovascular invasion), N category (classified by lymph node metastasis), and M category (classified by distant metastasis) were significantly associated with worse survival, and their survival curves were consistent with previous reports. Interestingly, the survival of patients in stage II (localized invasive tumors) was worse than that of patients in stage IIIa (one regional lymph node metastasis), although the difference did not reach statistical significance (*p* = 0.47). These inverse survival results were also observed in the original report of the TNM staging system for EMPD (8). The exact mechanisms of this inversion is still unclear but this is also noted in malignant melanoma (41, 42). EMPD and melanoma exhibit a similar invasion process (first arising in the epidermis, horizontally spreading, and later invading vertically into the dermis with the destruction of basal membrane). One possible explanation is the hematogenous metastasis, however, more data is required to test this hypothesis.

## Conclusion

We retrospectively reviewed 23 years of data of 217 patients with EMPD. Most patients (n = 198, 91.2%) were candidates for curative surgery. Tumor involvement in boundary areas was a major risk factor for incomplete excision, local recurrence, and poor survival outcomes. However, incomplete removal of primary tumors was not significantly associated with poor prognosis. A less invasive surgical approach for preserving anogenital and urinary functions may be acceptable as the first-line treatment for resectable EMPD.

## Data Availability Statement

The raw data supporting the conclusions of this article will be made available by the authors, without undue reservation.

## Ethics Statement

The studies involving human participants were reviewed and approved by Kyushu University Hospital. The patients/participants provided their written informed consent to participate in this study.

## Author Contributions

HH, TI, and YK-I participated in manuscript preparation. TI designed the methodology. HH participated in data analysis and figure preparation. HH and YK-I collected the detailed information of the patients. TI and MF reviewed and revised the manuscript. All authors contributed to the article and approved the submitted version.

## Conflict of Interest

The authors declare that the research was conducted in the absence of any commercial or financial relationships that could be construed as a potential conflict of interest.

## Abbreviations

EMPD, extramammary Paget’s disease; TNM, tumor, node, and metastasis; DSS, disease-specific survival; SLNB, sentinel lymph node biopsy; CLND, completion lymph node dissection.
