# Supplementary material for: Mucosal Invasion, but Not Incomplete Excision, Has Negative Impact on Long-Term Survival in Patients With Extramammary Paget’s Disease
Source: Front Oncol. 2021 Apr 15;11:642919. doi: 10.3389/fonc.2021.642919 (PMC8082157; doi:10.3389/fonc.2021.642919)
Supplement: Supplementary file 1 [file Table_1.docx]

Supplementary Material

# Supplementary Tables

Supplementary Table 1. Patterns of initial treatment

| Treatment | n (%) |
| --- | --- |
| Primary lesion  Surgical excision  Curative surgery  Additional excision  Palliative surgery  No excision | 204 (94.0)  200 (92.2)  7 (3.4)  4 (1.8)  13 (6.0) |
| Regional LNs  No lymphadenopathy + SLNB  LN metastasis present  No LN metastasis  Biopsy of lymphadenopathy  LN metastasis present  No LN metastasis  CLND | 33 (15.2)  8  25  19 (8.8)  9  10  18 (8.3) |
| Chemotherapy and/or targeted therapy | 6 (2.8) |
| Radiation therapy | 7 (3.2) |
| Palliative care or observation | 2 (0.9) |
| Total | 217 (100) |

LN, lymph node; SLNB, sentinel lymph node biopsy; CLND, completion lymph node dissection.

Supplementary Table 2. Patients with local recurrence

| Case | Age, y | Sex | Tumor site | Boundary area involvement | Stage at initial treatment | Excision margin, cm | Surgical margin status | Additional excision | RFS, mo^†^ | Treatment to recurrence | FU, mo^‡^ | Status at last FU |
| --- | --- | --- | --- | --- | --- | --- | --- | --- | --- | --- | --- | --- |
| 1 | 70 | F | G | + | 0 | 3 | N | Not done | 93.4 | Excision | 218.5 | Alive |
| 2 | 68 | F | G | + | 0 | 2 | N | Not done | 157.0 | Excision | 170.2 | Alive |
| 3 | 69 | F | G | + | 0 | 2 | P | Not done | 44.3 | Excision | 81.2 | DAC |
| 4 | 82 | F | G | + | 0 | 2 | N | Not done | 26.4 | Topical imiquimod | 40.8 | DAC |
| 5 | 67 | F | G | + | 0 | 2 | P | Done | 102.4 | Excision | 127.8 | Alive |
| 6 | 64 | F | G | + | 0 | 2 | P | Not done | 25.7 | Topical imiquimod | 198.3 | Alive |
| 7 | 63 | F | G | + | 0 | 2 | P | Not done | 21.1 | Excision | 173.5 | Alive |
| 8 | 66 | M | G | + | I | 3 | N | Not done | 246.3 | Radiation therapy | 256.5 | Alive |
| 9 | 71 | M | G | + | I | 2 | P | Not done | 12.4 | Excision | 173.7 | Alive |
| 10 | 65 | F | G | + | I | 1 | P | Not done | 10.0 | Excision | 55.7 | DPD |
| 11 | 34 | F | G | + | I | 1 | N | Not done | 18.1 | Excision | 21.2 | Alive |
| 12 | 82 | F | G + A | + | I | 2 | N | Not done | 30.9 | Excision | 44.7 | Alive |
| 13 | 76 | F | G + A | + | IV | 0.5 | P | Not done | 2.4 | Radiation therapy | 12.7 | DPD |

† Time from the date of the initial treatment to the date of death due to extramammary Paget’s disease or local recurrence.

‡ Time from the date of the first histological examination to the date of death due to extramammary Paget’s disease or the last follow-up prior to October 31, 2020.

Y, year; M, male; F, female; RFS, relapse-free survival; P, positive; N, negative; mo, month; G, genital; A, anal; CSE, curative surgical excision; FU, follow-up; DAC, death from another cause, DPD; death from Paget’s disease.

Supplementary Table 3. Multivariate Cox proportional hazard analyses in 65 patients with boundary area involvement for disease-specific survival

| Variable | Univariate analysis | | |  | Multivariate analysis | | |
| --- | --- | --- | --- | --- | --- | --- | --- |
|  | HR | 95% CI | *P*-value |  | HR | 95% CI | *P*-value |
| Sex, male | 5.86 | 1.50-22.86 | **0.011** |  | 0.0043 | 0.000039-0.48 | **0.024** |
| Age (year)^†^ | 1.01 | 0.94-1.08 | 0.84 |  | 1.10 | 0.99-1.23 | 0.082 |
| Perianal lesion | 0.54 | 0.068-4.33 | 0.56 |  | 0.74 | 0.036-15.17 | 0.85 |
| Tumor thickness > 4 mm | 27.63 | 6.44-118.67 | **< 0.0001** |  | 43.61 | 2.32-819.81 | **0.012** |
| Incomplete excision | 0.26 | 0.054-1.22 | 0.088 |  | 3.11 | 0.31-31.44 | 0.34 |
| Regional LN metastasis | 22.71 | 4.75-108.67 | **< 0.0001** |  | 1279.54 | 13.00-126020.38 | **0.0023** |

Significant values are shown in boldface.

† Continuous variable.

HR, hazard ratio; CI, confidence interval; LN, lymph node.
